# Discordance in *Mycobacterium tuberculosis* Rifampin Susceptibility

**DOI:** 10.3201/eid1803.111357

**Published:** 2012-03

**Authors:** Ameeta S. Kalokhe, Majid Shafiq, James C. Lee, Beverly Metchock, James E. Posey, Susan M. Ray, Albert Anderson, Yun F. Wang, Minh Ly T. Nguyen

**Affiliations:** Emory University School of Medicine, Atlanta, Georgia, USA (A.S. Kalokhe, M. Shafiq, J.C. Lee, S.M. Ray, A. Anderson, Y.F. Wang, M.T. Nguyen);; Centers for Disease Control and Prevention, Atlanta (B. Metchock, J.E. Posey, M.T. Nguyen)

**Keywords:** multidrug-resistant tuberculosis, molecular diagnostic testing, microbial sensitivity tests, Mycobacterium tuberculosis, tuberculosis and other mycobacteria, rifampin susceptibility, antimicrobial resistance, MDR TB, TB

**To the Editor:** Multidrug-resistant tuberculosis (MDR TB), i.e., TB resistant to at least the 2 most effective first-line antituberculous drugs (isoniazid [INH] and rifampin [RIF]), is increasing globally. World Health Organization estimations of 390,000–510,000 new MDR TB cases and 150,000 related deaths in 2008 highlight the need for timely drug susceptibility testing and improved therapies (*1*). Although novel rapid drug susceptibility testing tools are increasingly available, their clinical applicability is unsettled. We report a patient with pulmonary TB relapse with discordant genotypic and in vitro phenotypic drug susceptibility testing results associated with a mutation outside the RIF resistance determining region (RRDR) of the *rpoB* gene.

In August 2009, a 45-year-old homeless woman with AIDS (CD4^+^ T-cell count 3 cells/mm^3^) and a history of substance abuse sought care for fever, night sweats, weight loss, and cough (Table A1). Pulmonary TB had been diagnosed in June 2008. At that time, she received, by directly observed therapy, 6 weeks of INH, RIF, pyrazinamide (PZA), and ethambutol (EMB) through the local health department and was switched to RIF, PZA, and EMB on week 7 after the isolate was determined by liquid culture with BD BACTEC MGIT 960 Mycobacterial Testing System (BD Diagnostics, Sparks, MD, USA) to be INH resistant. During that period, she resided in American Lung Association–supported housing for TB patients and had 97% medication adherence by dose count. Her condition clinically improved, infiltrates completely resolved according to chest radiograph, 12 sputum inductions failed to yield sufficient material for analysis, and she began highly active antiretroviral therapy (HAART). In December 2008, however, because of crack cocaine use and belligerent behavior, she lost housing privileges. Caseworkers could not locate her to complete the 9-month planned directly observed therapy.

The woman was hospitalized in January and again in February 2009 with dyspnea and off medications. In both instances, chest radiographs showed no new changes, sputum specimens were negative for acid-fast bacilli (AFB) by microscopy and culture, and she was treated for presumptive *Pneumocystis* pneumonia and showed clinical improvement.

In August 2009, she was readmitted with cough and new cavitation on chest radiograph. Chest computed tomographic scan demonstrated right upper lobe infiltrates, bilateral lower lobe cavitation, and hilar and mediastinal lymphadenopathy. Sputum AFB smear was positive (graded 4+), and nucleic acid amplification (Amplified Mycobacterium Direct Test; Gen-Probe, San Diego, CA, USA) was positive for *Mycobacterium tuberculosis* complex. INH, RIF, PZA, and EMB, along with moxifloxacin (MXF) and amikacin (AMK), were initiated in accordance with 2003 national TB treatment guidelines for possible MDR TB (*2*). Shortly thereafter, a line probe assay (GenoType MTBDR*plus*; HAIN Lifescience, Nehren, Germany) performed by Southeastern National TB Center (Gainesville, FL, USA) on the culture of the sputum specimen obtained at admission indicated an *inhA* point mutation but no mutation in the RRDR region of the *rpoB* gene, which suggested that the isolate was INH resistant but RIF susceptible. AMK was discontinued, and the patient was discharged with RIF, PZA, EMB, and MXF.

One week later, drug susceptibility testing (BD BACTEC MGIT 960 System) results from the state mycobacteriology laboratory demonstrated that the *M. tuberculosis* isolate was resistant to INH and RIF. The patient was readmitted to resume injectable aminoglycoside therapy. After 5 weeks, sputum culture became negative, clinical and radiographic improvement was apparent, and HAART was reinitiated. She completed 2 months’ INH/PZA/EMB/MXF/AMK inpatient therapy and was discharged to complete 6 additional months of PZA/EMB/MXF and streptomycin followed by 16 months of PZA/EMB/MFX. With HAART, her plasma HIV RNA viral load became undetectable, but her CD4 count remained low (9 cells/mm^3^). She died from a motor vehicle accident 10 months after recurrent TB was diagnosed.

In this patient, RIF resistance was not predicted by line probe assay but was identified phenotypically by an automated system (BD BACTEC MGIT 960 System) that continuously monitors for growth and detection of mycobacteria. Through genotyping and DNA sequencing of the 2008 and 2009 *M. tuberculosis* isolates, the Mycobacteriology Laboratory Branch at the Centers for Disease Control and Prevention (Atlanta, GA, USA) established that the 2009 infection was a relapse, not re-infection, and confirmed the *inhA* mutation in both isolates. Using primers extending beyond the RRDR (the *rpoB* region surveyed by rapid molecular tests and responsible for >95% of RIF resistance mutations) the laboratory identified a novel *rpoB* gene mutation at codon 480 (ACC→AAC; Thr→Asn) and another previously described (*3**–**5*) mutation at codon 176 (GTC→TTC; Val→Phe) in the 2009 isolate, which has been implicated in RIF resistance. The role of the T480N mutation in RIF resistance is being investigated.

This case demonstrates the limitations of rapid molecular drug susceptibility testing (*6*). Rapid molecular diagnostics are valuable adjuncts to conventional phenotypic testing because they can quickly confirm clinically suspected MDR TB and have high agreement with other genotypic and phenotypic methods (*7**–**10*). However, they should not supplant phenotypic testing, and clinicians should understand their limitations. When rapid molecular tests are negative but suspicion for MDR TB is high, MDR TB treatment should be continued until phenotypic susceptibility results are available. DNA sequencing may be best suited for evaluating suspected drug-resistant *M. tuberculosis* isolates with discordant results for phenotypic susceptibility and rapid molecular testing.

## Appendix

**Table A1:** AFB culture sequence of patient with discordant in *Mycobacterium tuberculosis* RF susceptibility*

Date	2008					2009										
	May 13	Jun 2	Dec 28	Jan 31		Feb 3	Feb 24	Aug 3	Aug 4	Aug 5	Aug 19	Sep 10	Sep 11	Oct 5	Oct 7	Oct 12
Specimen type	Sp × 3	BAL	Sp × 3	Sp		Sp × 2	Sp × 3	Blood	Sp	Sp	Sp	Blood	Sp	Sp	Sp	Sp
AFB smear (MTD)	–	4+ (MTD+)	–	–		–	–	NA	4+ (MTD+)	4+ (MTD+)	1+	NA	1+ (MTD+)	–	–	–
AFB culture	–	*M. tuberculosis*	–	*M. avium-intraceullulare*		–	–	*M. tuberculosis*	*M. tuberculosis*	*M. tuberculosis*	*M. tuberculosis, M. avium-intraceullulare*	–	–	–	–	–
Drug susceptibility (MGIT 960)		INH resistant (0.2 μg/mL); RIF susceptible; EMB susceptible; STR susceptible							INH resistant (0.1 μg/mL) RIF resistant EMB susceptible STR susceptible		INH resistant (0.2 μg/mL); RIF resistant; EMB susceptible; STR susceptible					
Molecular resistance testing		Sequencing: *inhA* mutation associated with INH resistance									HAIN test: i*nhA* point mutation; no *rpoB* mutation detected	CDC genotyping: *inhA* mutation associated with INH resistance + novel *rpoB* mutation (2009 Sep 9)				
Drug treatment		INH/RIF/PZA/EMB initiated (2008 Jun 5)	6 mos. RIF/PZA/ EMB completed (2008 Dec 12)						INH/RIF/PZA/ EMB, AMK, MXF initiated (2009 Aug 4)		AMK stopped (2009 Sep 1)	AMK resumed (2009 Sep 9)				

*RIF, rifampin; AFB, acid-fast bacilli; sp, sputum; BAL, bronchoalveolar lavage; MTD, *Mycobacterium tuberculosis* Direct Test (Gen-Probe, San Diego, CA, USA); –, negative; +, positive; NA, not applicable; MGIT, BACTEC Mycobacterium Growth Indicator Testing (MGIT 960; Becton Dickinson, Franklin Lakes, NJ, USA); INH, isoniazid; RIF, rifampin; EMB, ethambutol; INH, isoniazid; STR, streptomycin; HAIN, GenoType MTBDR plus line probe assay (HAIN Lifescience, Nehren, Germany); CDC, Centers for Disease Control and Prevention (Atlanta, GA, USA); PZA, pyrazinamide; AMK, amikacin; MXF, moxifloxacin.

## References

[1] World Health Organization. Multidrug and extensively drug-resistant TB (M/XDR-TB): 2010 global report on surveillance and response. Report no. WHO/HTM/TB/2010.3. Geneva: The Organization; 2010.

[2] American Thoracic Society, Centers for Disease Control and Prevention, Infectious Diseases Society of America. Treatment of tuberculosis [Erratum in MMWR Morb Mortal Wkly Rep. 2005;53:1203]. MMWR Recomm Rep. 2003;52(RR-11):1–77. 12836625

[3] Heep M, Rieger U, Beck D, Lehn N. Mutations in the beginning of the *rpoB* gene can induce resistance to rifamycins in both *Helicobacter pylori* and *Mycobacterium tuberculosis.* Antimicrob Agents Chemother. 2000;44:1075–7. 10.1128/AAC.44.4.1075-1077.2000 10722516 PMC89817

[4] Tan Y, Hu Z, Zhao Y, Cai X, Luo C, Zou C, The beginning of the *rpoB* gene in addition to the RRDR might be needed for identifying RIF/Rfb cross resistance in multidrug-resistant *Mycobacterium tuberculosis* isolates from southern China. J Clin Microbiol. 2012;50:81–5. 10.1128/JCM.05092-11 22075601 PMC3256682

[5] Heep M, Brandstatter B, Rieger U, Lehn N, Richter E, Rusch-Gerdes S, Frequency of *rpoB* mutations inside and outside the cluster I region in rifampin-resistant clinical *Mycobacterium tuberculosis* isolates. J Clin Microbiol. 2001;39:107–10. 10.1128/JCM.39.1.107-110.2001 11136757 PMC87688

[6] Van Deun A, Barrera L, Bastian I, Fattorini L, Hoffmann H, Kam KM, *Mycobacterium tuberculosis* strains with highly discordant rifampin susceptibility test results. J Clin Microbiol. 2009;47:3501–6. 10.1128/JCM.01209-09 19759221 PMC2772627

[7] Ling DI, Zwerling AA, Pai M. GenoType MTBDR assays for the diagnosis of multidrug-resistant tuberculosis: a meta-analysis. Eur Respir J. 2008;32:1165–74. 10.1183/09031936.00061808 18614561

[8] Morgan M, Kalantri S, Flores L, Pai M. A commercial line probe assay for the rapid detection of rifampicin resistance in *Mycobacterium tuberculosis*: a systematic review and meta-analysis. BMC Infect Dis. 2005;5:62. 10.1186/1471-2334-5-62 16050959 PMC1185540

[9] Boehme CC, Nabeta P, Hillemann D, Nicol MP, Shenai S, Krapp F, Rapid molecular detection of tuberculosis and rifampin resistance. N Engl J Med. 2010;363:1005–15. 10.1056/NEJMoa0907847 20825313 PMC2947799

[10] Bravo LT, Tuohy MJ, Ang C, Destura RV, Mendoza M, Procop GW, Pyrosequencing for rapid detection of *Mycobacterium tuberculosis* resistance to rifampin, isoniazid, and fluoroquinolones. J Clin Microbiol. 2009;47:3985–90. 10.1128/JCM.01229-09 19846642 PMC2786679

